# Supplementary material for: A Flow Cytometry-Based Examination of the Mouse White Blood Cell Differential in the Context of Age and Sex
Source: Cells. 2024 Sep 20;13(18):1583. doi: 10.3390/cells13181583 (PMC11430320; doi:10.3390/cells13181583)
Supplement: Supplementary file 1 [file cells-13-01583-s001.zip › cells-3160792-supplementary.pdf]

Supplementary Material

Table S1: List of reagents / materials / equipment

Reagent / material / equipment	Company	Cat. number
Sterile Disposable Needle 25G	Berpu Medical	BP2530430
Micro test tube EDTA K3E, 1.3 ml, screw cap, EU	Sarstedt	41.3395.005
EDTA (diluted 16 mg/ml)	Roth	8043.1
Pappenheim staining kit	Morphisto	11103
5 ml Polystyrene Round-Bottom Tube	Falcon	352052
FCA Buffer	NA, self-made: Filtered DPBS, 1 % fetal calf serum, 2 mM EDTA, 0.05% sodium acid	
Red Blood Cell Lysis Buffer	c.c.pro GmbH	PL-29-L
MACS Quant Analyzer 16	Miltenyi Biotec	130-109-803
MACS Comp Bead Kit, anti-REA	Miltenyi Biotec	130-104-693
REA Control Antibody, human IgG1, REAfinity™, VioBlue	Miltenyi Biotec	130-113-454
REA Control Antibody, human IgG1, REAfinity™, VioGreen	Miltenyi Biotec	130-113-456
REA Control Antibody, human IgG1, REAfinity™, FITC	Miltenyi Biotec	130-113-449
REA Control Antibody, human IgG1, REAfinity™, PE	Miltenyi Biotec	130-113-450
REA Control Antibody, human IgG1, REAfinity™, PE-Vio770	Miltenyi Biotec	130-113-452
REA Control Antibody, human IgG1, REAfinity™, APC	Miltenyi Biotec	130-113-446
REA Control Antibody, human IgG1, REAfinity™, APC-Vio770	Miltenyi Biotec	130-113-447
Propidium Iodide Solution	Miltenyi Biotec	130-093-233
CD45 Antibody, anti-mouse, REAfinity™, VioBlue	Miltenyi Biotec	REA737<br>130-110-664
IgG1 Antibody, anti-mouse, REAfinity™, VioBlue	Miltenyi Biotec	REA1017<br>130-117-100
CD8b Antibody, anti-mouse, REAfinity™, VioGreen	Miltenyi Biotec	REA793<br>130-111-717
MHC Class II Antibody, anti-mouse, REAfinity™, VioGreen	Miltenyi Biotec	REA813<br>130-112-395
CD11b Antibody, anti-mouse, REAfinity™, VioGreen	Miltenyi Biotec	REA592<br>130-113-811

Ly-6C Antibody, anti-mouse, REAfinity™, VioGreen	Miltenyi Biotec	REA796<br>130-111-922
CD62L Antibody, anti-mouse, REAfinity™, FITC	Miltenyi Biotec	REA828<br>130-112-835
Ly-6C Antibody, anti-mouse, REAfinity™, FITC	Miltenyi Biotec	REA796<br>130-111-915
Siglec-F Antibody, anti-mouse, REAfinity™, FITC	Miltenyi Biotec	REA798<br>130-112-336
CD3 Antibody, anti-mouse, REAfinity™, FITC	Miltenyi Biotec	REA641<br>130-119-798
IgD Antibody, anti-mouse, REAfinity™, FITC	Miltenyi Biotec	REA772<br>130-111-495
FcεRIα Antibody, anti-mouse, REAfinity™, VioBright	Miltenyi Biotec	REA1079<br>130-118-903
CD25 Antibody, anti-mouse, REAfinity™, PE	Miltenyi Biotec	REA568<br>130-120-766
CD335 (NKp46) Antibody, anti-mouse, REAfinity™, PE	Miltenyi Biotec	REA815<br>130-112-358
CD11b Antibody, anti-mouse, REAfinity™, PE	Miltenyi Biotec	REA592<br>130-113-806
CD317 (PDCA-1) Antibody, anti-mouse, REAfinity™, PE	Miltenyi Biotec	REA818<br>130-112-377
CD95 (FAS) Antibody, anti-mouse, REAfinity™, PE	Miltenyi Biotec	REA453<br>130-119-657
Sca-1 Antibody, anti-mouse, REAfinity™, PE-Vio770	Miltenyi Biotec	REA422<br>130-123-891
CD4 Antibody, anti-mouse, REAfinity™, PE-Vio770	Miltenyi Biotec	REA604<br>130-123-894
CD27 Antibody, anti-human/mouse, REAfinity™, PE-Vio770	Miltenyi Biotec	REA499<br>130-113-641
CD11c Antibody, anti-mouse, REAfinity™, PE-Vio770	Miltenyi Biotec	REA754<br>130-110-840
CD200R3 Antibody, anti-mouse, REAfinity™, PE-Vio770	Miltenyi Biotec	REA128<br>130-102-248
F4/80 Antibody, anti-mouse, REAfinity™, PE-Vio770	Miltenyi Biotec	REA126<br>130-118-459
CD314 (NKG2D) Antibody, anti-mouse, REAfinity™, APC	Miltenyi Biotec	REA1175<br>130-121-100
CD3 Antibody, anti-mouse, REAfinity™, APC	Miltenyi Biotec	REA641<br>130-122-943
Ly-6G Antibody, anti-mouse, REAfinity™, APC	Miltenyi Biotec	REA526<br>130-120-803

CD117 Antibody, anti-mouse, REAfinity™, APC	Miltenyi Biotec	REA791<br>130-111-694
CD4 Antibody, anti-mouse, REAfinity™, APC	Miltenyi Biotec	REA604<br>130-116-487
NK1.1 Antibody, anti-mouse, REAfinity™, APC	Miltenyi Biotec	REA1162<br>130-120-507
CD138 Antibody, anti-mouse, REAfinity™, APC	Miltenyi Biotec	REA104<br>130-122-945
IgM Antibody, anti-mouse, REAfinity™, APC	Miltenyi Biotec	REA979<br>130-116-313
FoxP3 Antibody, anti-mouse, REAfinity™, APC	Miltenyi Biotec	REA788<br>130-111-679
NK1.1 Antibody, anti-mouse, REAfinity™, APC-Vio770	Miltenyi Biotec	REA1162<br>130-120-510
CD3 Antibody, anti-mouse, REAfinity™, APC-Vio770	Miltenyi Biotec	REA641<br>130-119-793
CD19 Antibody, anti-mouse, REAfinity™, APC-Vio770	Miltenyi Biotec	REA749<br>130-112-038
CD44 Antibody, anti-mouse, REAfinity™, APC-Vio770	Miltenyi Biotec	REA664<br>130-118-695
FoxP3 Staining Buffer Set	Miltenyi Biotec	130-093-142

Figure S1: FMO controls for the gating strategies in the main text

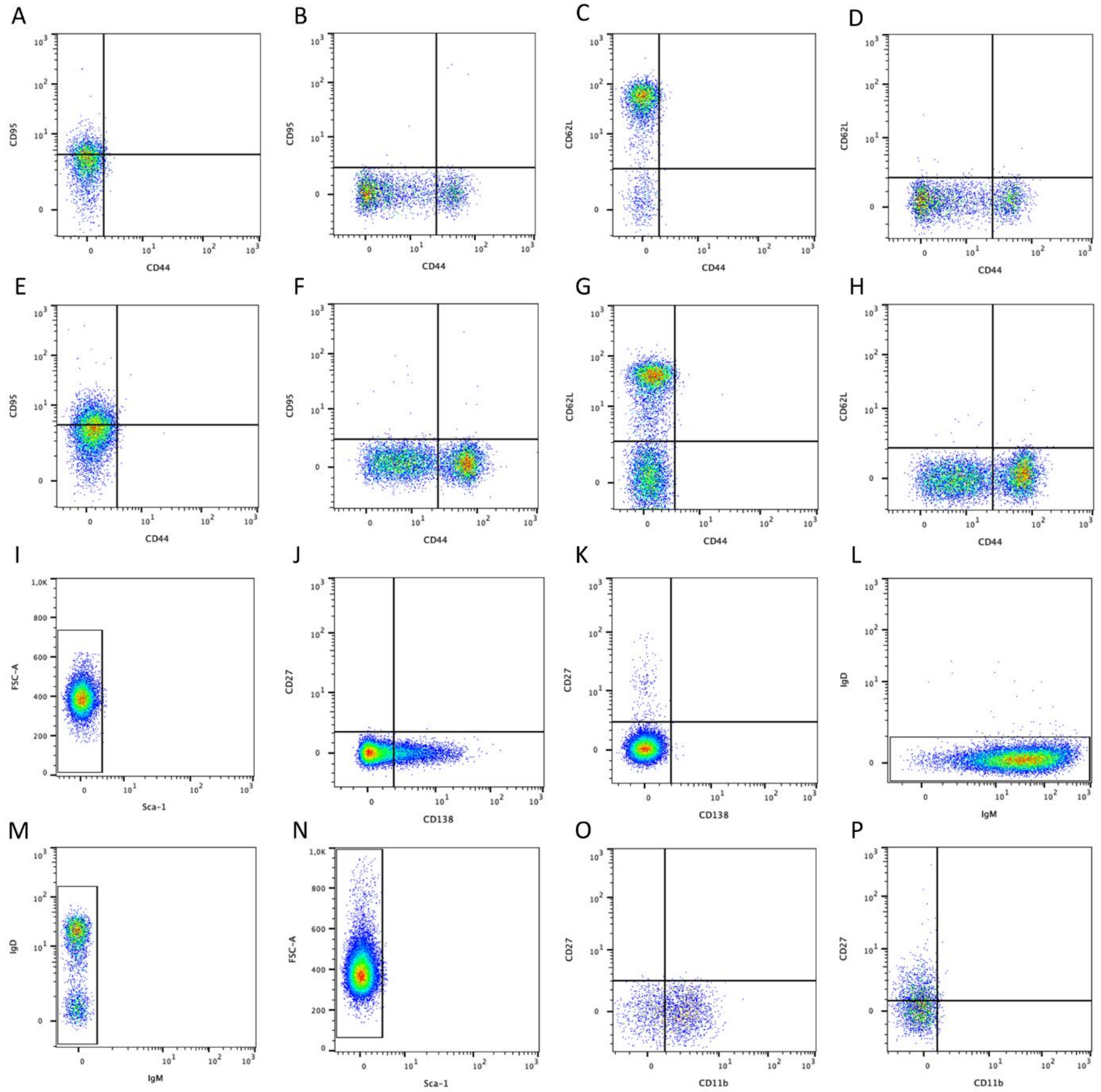

The FMOs were done by replacing the antibody with 0.3 μg of REA Control Antibody for the same fluorochrome in the antibody staining mastermix.

A FMO for CD44 on CD8⁺ T cells, Fig. 3A/5 **B** FMO for CD95 on CD8⁺ T cells, Fig. 3A/5 **C** FMO for CD44 on CD8⁺ T cells, Fig. 3A/6 **D** FMO for CD62L on CD8⁺ T cells, Fig. 3A/6 **E** FMO for CD44 on CD4⁺ T cells, Fig. 3A/7 **F** FMO for CD95 on CD4⁺ T cells, Fig. 3A/7 **G** FMO for CD44 on CD4⁺ T cells, Fig. 3A/8 **H** FMO for CD62L on CD4⁺ T cells, Fig. 3A/8 **I** FMO for Sca-1 on T cells, Fig 3C1+2 **J** FMO for CD27 on B cells, Fig. 4A/3 **K** FMO for CD138 on B cells, Fig. 4A/3 **L** FMO for IgD on B cells, Fig. 4A/4 **M** FMO for IgM on B cells, Fig. 4A/4 **N** FMO For Sca-1 on B cells, Fig. 4A/6 **O** FMO for CD27 on NK cells, Fig. 5A/3 **P** FMO for CD11b on NK cells, Fig. 5A/3

Table S2: Mean values and ranges (min-max)

This table provides reference values and ranges for the leukocyte types that are discussed in the manuscript.

Leukocyte subset	3 Mo M	3 Mo F	24 Mo M	24 Mo F
Lymphocytes ($CD45^+$)	70.5 (62.4-74.2)	78.07 (72.9-81.8)	63.7 (58-71.5)	61.42 (52-72)
T cells ($CD45^+$)	20.63 (18.2-25)	25.83 (22-30)	11.85 (7.2-18)	11.44 (8.1-13.6)
B cells ($CD45^+$)	44.75 (38-48.4)	47.37 (36.9-52.5)	48.3 (42-57)	46 (36-55)
NK cells ($CD45^+$)	4.66 (3.6-7.1)	4.87 (3.7-7.5)	2.04 (0.96-3.15)	2.66 (0.97-4.98)
NKT cells ($CD45^+$)	2.1 (1.1-4)	3.3 (0.6-6.3)	0.9 (0.45-2.1)	0.89 (0.2-2.36)
$CD4^+$ T cells ($CD45^+$)	51.72 (48-54.8)	54.65 (49.4-58.4)	29.5 (14.5-40)	30.33 (20-39)
$CD8^+$ T cells ($CD45^+$)	38.73 (35.3-42.2)	36.15 (31-44.5)	57.5 (40-81)	57.1 (52-65)
$CD25^+$ T reg ($CD4^+$)	6.75 (4.9-10)	5.9 (3.5-7.6)	8.82 (6.5-11.5)	8.72 (5.4-13)
Monocytes ($CD45^+$)	10.67 (7.6-13.8)	8.55 (7-9.6)	13.82 (8.4-21.2)	16.03 (10.3-22.8)
Macrophages ($CD45^+$)	0.8 (0.07-3.5)	2.76 (0.2-5.65)	0.28 (0-0.8)	0.55 (0.07-1.34)
Dendritic cells ($CD45^+$)	0.69 (0.6-1.15)	0.65 (0.3-1.1)	0.39 (0.2-0.5)	0.96 (0.65-1.5)
Mast cells ($CD45^+$)	0.04 (0-0.18)	0.08 (0-0.15)	0.01 (0-0.02)	0.02 (0-0.065)
Granulocytes ($CD45^+$)	16.21 (12.1-25.4)	9.66 (7.2-12.34)	20.88 (18.6-24.8)	18.7 (9.2-28.4)
Neutrophils ($CD45^+$)	12.36 (8.8-21.7)	5.98 (4.9-8.6)	17.24 (13-20.8)	15.17 (6.3-23.7)
Eosinophils ($CD45^+$)	3.05 (2.5-3.64)	3.08 (1.7-4.08)	2.46 (1.5-3.6)	2.55 (1.1-3.6)
Basophils ($CD45^+$)	0.8 (0.7-1.01)	0.61 (0.38-0.8)	0.66 (0.1-1.5)	0.98 (0.7-1.6)

Figure S2: Residual values from panel 1-6

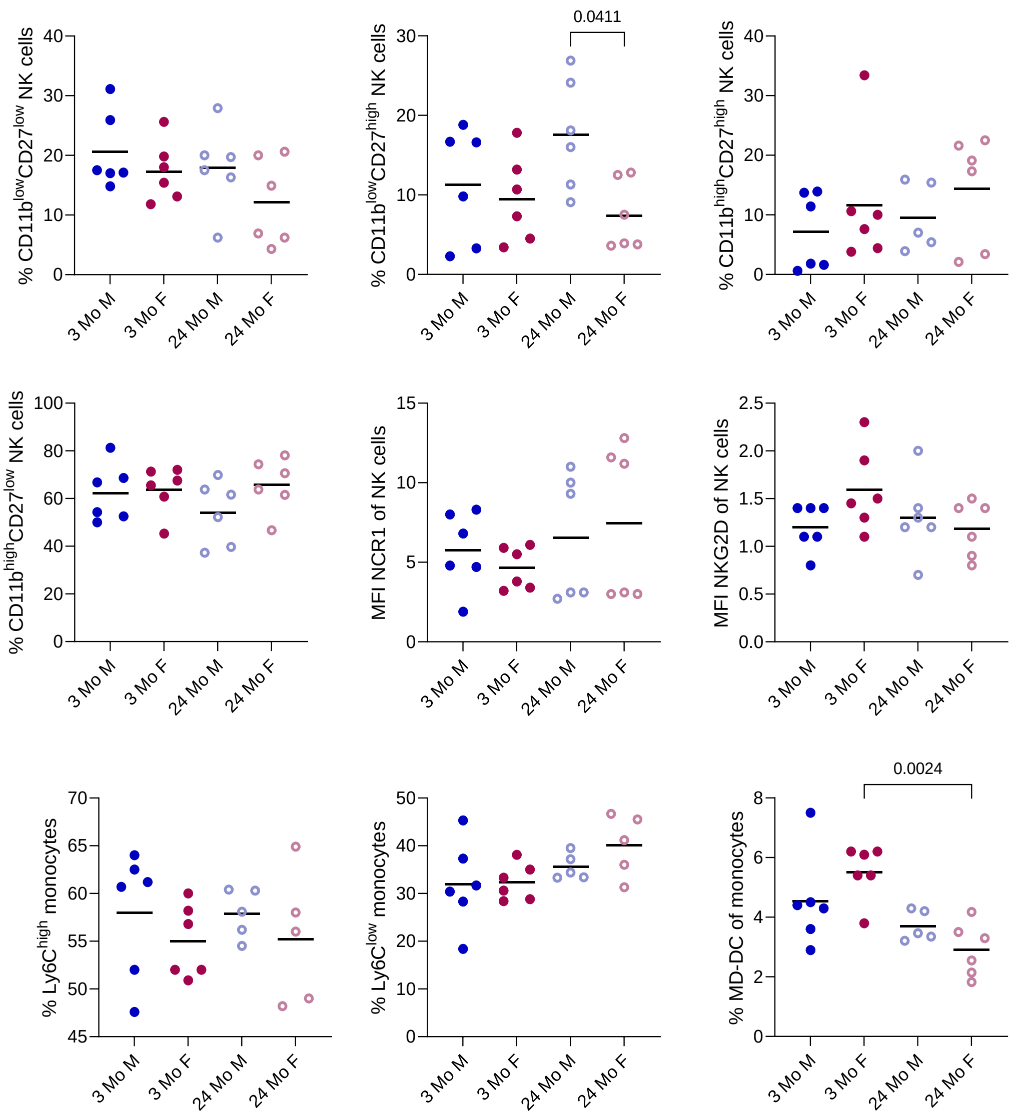

Values that were collected in the project but considered as less relevant or without large differences between the groups. Therefore, they are not shown in the main body of the manuscript.

Figure S3: T cell subpopulations in young and aged mice

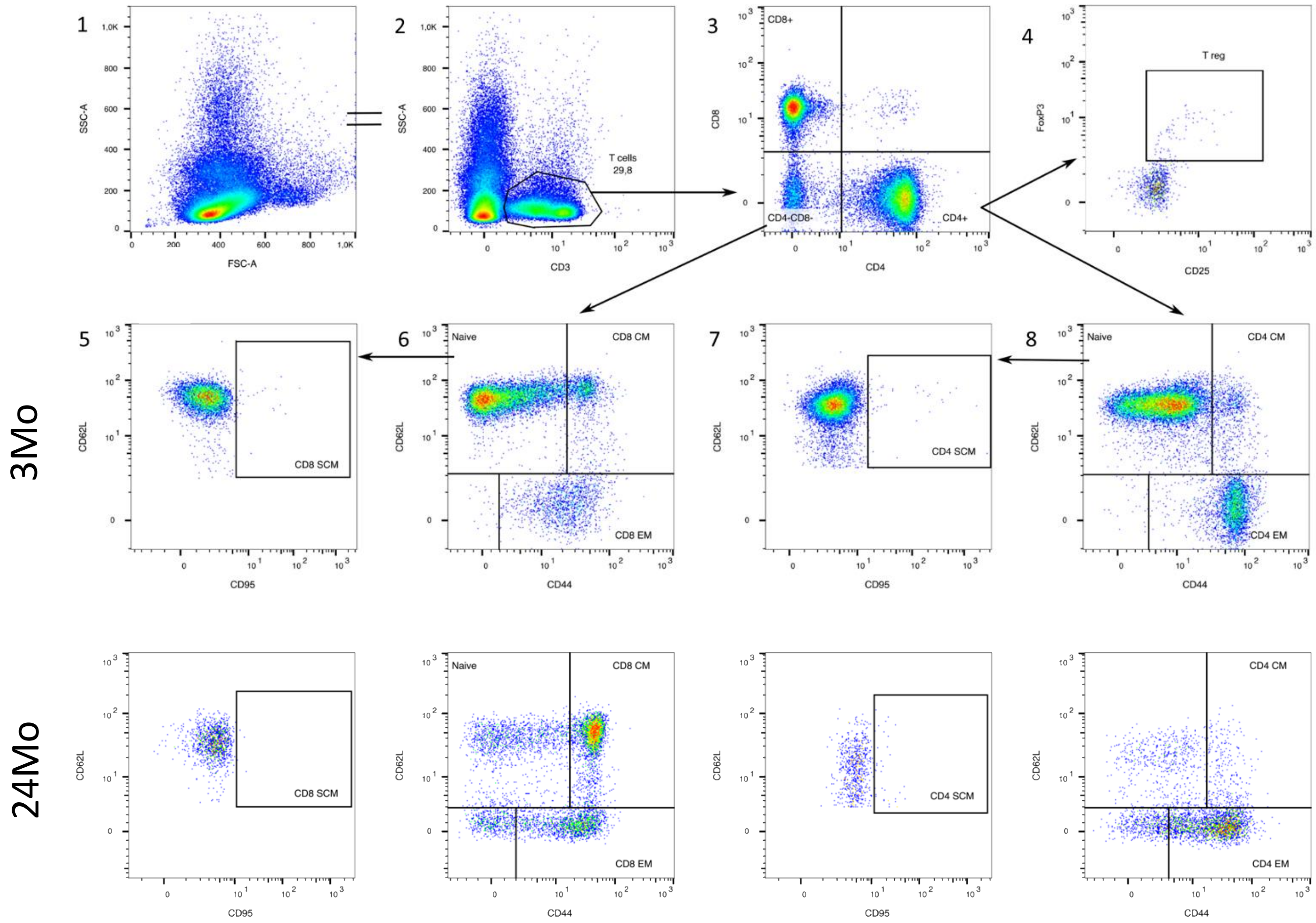

Gating of T cells including the different appearance of T cell subsets in young and aged mice. $CD3^+$ cells can be divided into $CD4^+$ T helper and $CD8^+$ cytotoxic T cells. Out of the $CD4^+$ T cells, $CD25^+FoxP3^+$ regulatory T cells can be distinguished. Both $CD4^+$ and $CD8^+$ T cells can be categorized into four different maturation stages: $CD44^{low}CD62L^+$ naive T cells, $CD62L^+CD44^{high}$ central memory T cells (CM), $CD62L^-CD44^+$ effector memory T cells (EM), $CD95^+CD44^{low}$ stem cell memory T cells (SCM). The plots of the T cell maturation stages look highly different between the 3-month-old and 24-month-old mouse groups. The CD4/CD8-plot and the FoxP3/CD25-plot only differ concerning the proportions and are therefore not shown for both age groups.

Figure S4: Variances in the appearance of the FSC-SSC- and the CD45-SSC-plot

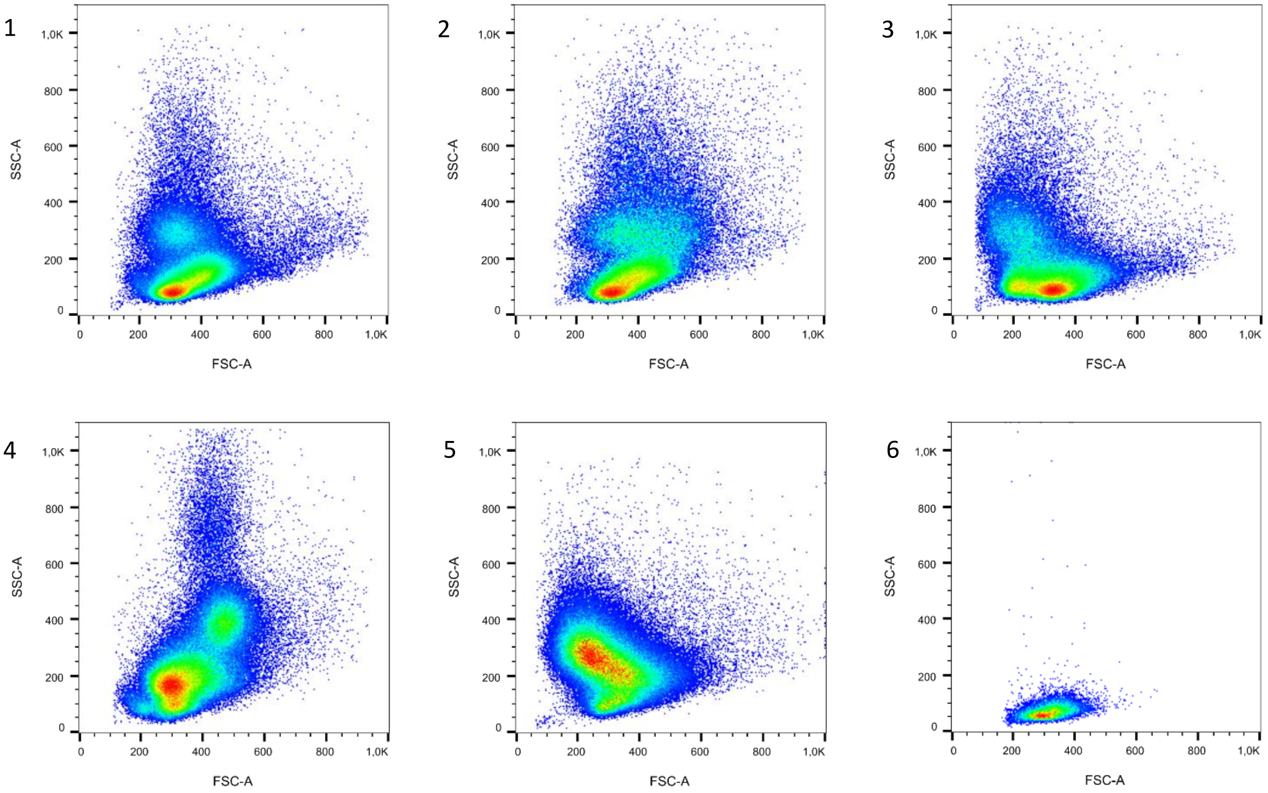

Individual appearance of the FSC-SSC-plot.
1-3 Physiological variances in mice that didn’t show deviating values concerning their blood count. **4+5** Mice whose blood count showed outliers that were not included in this study. **6** A sample that was permeabilized to stain FoxP3.

Figure S5: Blood smear

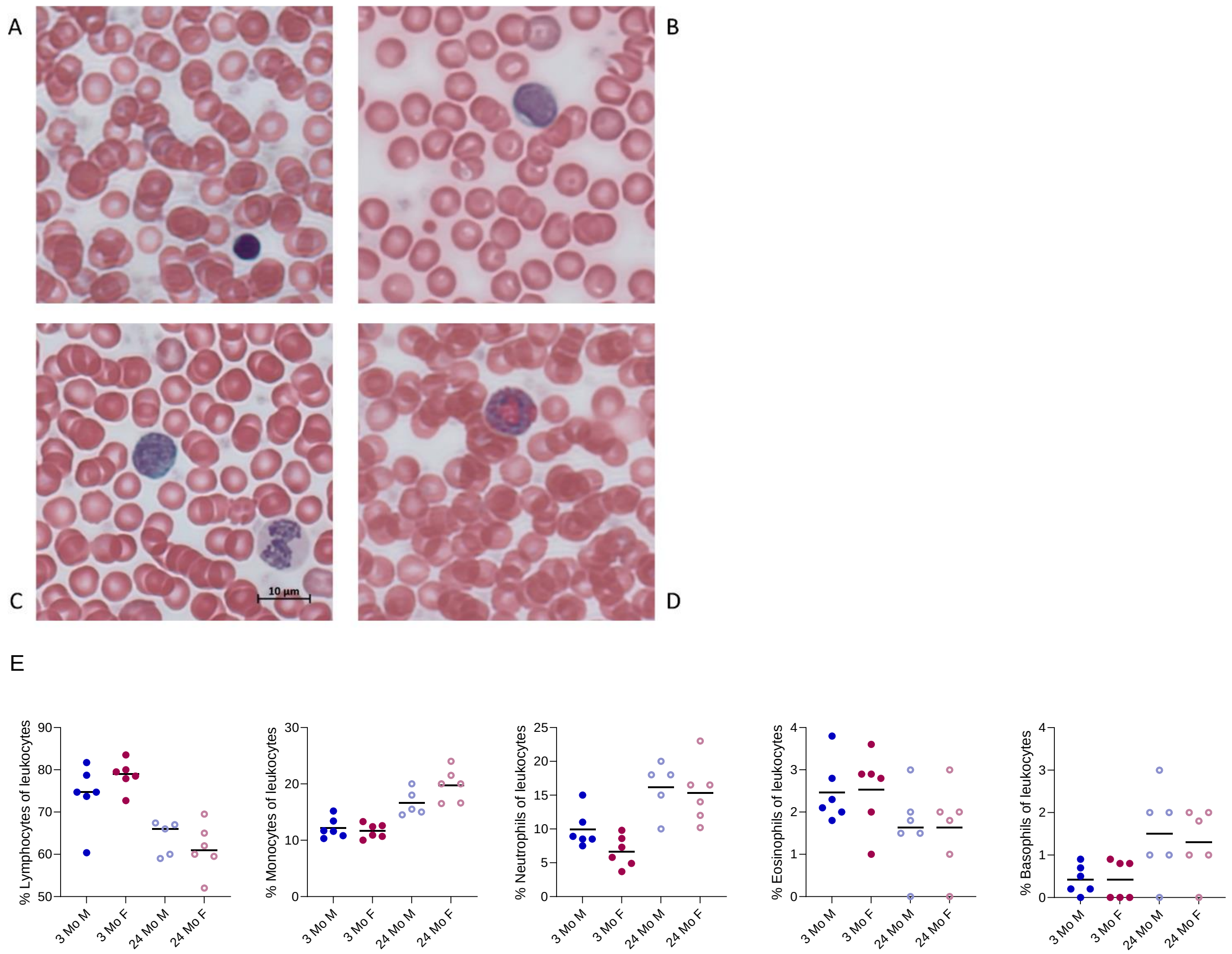

The pictures show leukocyte subsets in the murine blood smear.

A Lymphocyte, **B** Monocyte, **C** Basophil granulocyte (left) and neutrophil granulocyte (right), **D** Eosinophil granulocyte **E** Results from counting and differentiating 130 leukocytes under the microscope.
